# Accommodative Facility and Response Time before and after Computer Task of Varying Durations in Young Adults

**DOI:** 10.22599/bioj.295

**Published:** 2023-10-16

**Authors:** Asha Kaliugavaradhan, Dharani Ramamurthy

**Affiliations:** 1 SRM Institute of Science & Technology, IN

**Keywords:** monocular near accommodative facility, positive response time, negative response time, computer work, laptop viewing

## Abstract

**Aim:**

To investigate the changes in near accommodative facility and response time in young adults following computer work of 30 minutes and 1 hour in duration.

**Methods:**

A total of 50 young adults (37 females, 13 males) with mean age of 20.68 ± 1.33 years were included in this experimental study. Monocular near accommodative facility was measured using ±2.00 Dioptre Sphere (DS) flipper at 40 cm using the N6 (the smallest print size that can be read by an individual with normal visual acuity) target before and after two reading tasks. Both pre- and post-task measurements were video recorded using a smart phone and the number of cycles per minute, positive response time (time taken to stimulate accommodation), and negative response time (time taken to relax accommodation) were calculated from the video recording. Data were analysed using SPSS Version 22.0.

**Results:**

Out of the 50 participants, 29 were emmetropes (Mean SER: 0.16 ± 0.29 D), and 21 were myopes (Mean SER: –1.89 ± 1.16 D). The mean pre-task accommodative facility was 6.79 ± 3.52 cycles per minute, and the post-task accommodative facility was 6.25 ± 3.65 cycles per minute (p = 0.10) for the 30-minutes task and 5.76 ± 3.89 cycles per minute (p = 0.01) for 1-hour task. The mean pre-task positive response time was 2.87 ± 1.55 seconds, and the post-task positive response times for 30 minutes and 1 hour were 2.86 ± 1.67 seconds (p = 0.88) and 2.98 ± 2.33 seconds (p = 0.42), respectively. The mean pre-task negative response time was 8.77 ± 8.83 seconds, and the post-task negative response times for 30 minutes and 1 hour task were 11.83 ± 14.28 seconds (p = 0.16) and 14.72 ± 17.32 seconds (p = 0.03), respectively.

**Conclusion:**

Monocular near accommodative facility was significantly reduced, and negative response time was delayed following 1 hour of computer work.

## Introduction

The accommodative facility test is a tool for measuring the overall dynamics of accommodation in the clinic, because it is correlated with symptoms and near work duration (Iribarren, Fornaciari & Hung, 2001). Accommodative facility is a mandatory assessment in asthenopic subjects and in people who perform intense near work but are not symptomatic (Iribarren, Fornaciari & Hung, 2001).The prevalence of accommodative infacility was reported to be 7% and 10.7% in rural and urban areas of southern India, respectively, among schoolchildren aged 7–17 years (Hussaindeen et al., 2017). In the US, the prevalence of accommodative infacility was shown to be 1.5% in children aged 6–18 years (Scheiman et al., 1996). A prevalence of 2.5% in rural areas and 13.4% in urban areas of South Korea was reported among children aged between 9 and 13 years (Jang & Park, 2015; Shin, Park & Park, 2009). In Ghana, prevalence among children aged 13–17 years was reported to be 19% (Abdul-Kabir et al., 2014). In the age group of 18–38 years, the prevalence of accommodative infacility was reported to be 5.8% in Portugal and 10.3% in Spain (Franco et al., 2022; Montés-Micó, 2001).

School-aged children detected to have asthenopic symptoms performed significantly poorer in both monocular and binocular facility tests than asymptomatic children (Hennessey, Iosue & Rouse, 1984). Although the penetration of electronic devices was less several decades ago, reduced accommodative facility caused asthenopic symptoms without using e-devices, and those children who performed intense near work were more likely to develop symptoms. Response time is the time taken to achieve maximum accommodative response from onset of stimulus (Szostek et al., 2018). Positive response time and negative response time are the extent of time needed to stimulate and relax accommodation, respectively (Pandian et al, 2006).

Digital devices have become a ubiquitous part of life. All age groups have significantly increased their use of digital devices in recent years, making frequent daily use for both social and professional purposes the new norm. Technology use is increasing quickly across older age groups; the percentage of the population in the 75+ age group, classified as ‘recent internet users’ has increased twofold from 20% to 40%, and in the 65–74 age group, it has increased from 52.0% to 77.5%. In the US, 37% of adults older than 60 years use digital devices for five or more hours each day. Younger adults are more likely to use social media and multitask, with 87% of those in the 20–29 age range reporting using two or more digital devices at once. Digital eye strain (DES), often known as computer vision syndrome, consists of a range of ocular and visual symptoms, and estimates indicate that among computer users, it may be 50% or more prevalent (Sheppard & Wolffsohn, 2018). DES can be caused by using digital devices continuously for two hours, according to the American Optometric Association. Nonetheless, there has been a rise in the use of digital devices during the most recent new Coronavirus disease (COVID-19) outbreak. Since the beginning of the COVID-19 pandemic, working from home and attending classes online have become standard practices (Kaur et al., 2022).

Digital devices require the individual to rapidly alter their accommodation to fixate on the visual display unit and then relax accommodation for far objects so as to sustain a clear image. Prior research conducted to see the effect of near work on electronic devices on accommodative facility has given mixed evidence (Jiang & White, 1999; Golebiowski et al, 2020; Padavettan et al., 2021; Jaiswal et al., 2019; Park et al., 2014; Rosenfield et al., 2010). Monocular accommodative facility reduced after playing a game on a computer screen for 20 minutes in participants aged 21–27 years (Jiang & White, 1999). Binocular facility significantly reduced after one hour of smartphone reading and after 30 minutes of smartphone reading, as well as after 30 minutes of reading on a tablet, in participants aged 18–30 years (Golebiowski et al, 2020; Padavettan et al., 2021; Jaiswal et al., 2019). A decrease in monocular and binocular facility by 20% was observed after two hours of computer work and after 90 minutes computer work among young adults aged 20–30 years, although the change after 90 minutes was not statistically significant (Jaiswal et al., 2019). Conversely, after 30 minutes of viewing a film on a smartphone, neither binocular nor monocular facility had significantly changed among young adults aged 20–28 years (Park et al., 2014). Similarly, no change was observed in monocular facility after 25 minutes of reading from a computer; however, binocular facility increased in 22 (100%) visually normal participants (Rosenfield et al., 2010). From the earlier study results, it is evident that extended usage of electronic devices for one hour or more causes a decrease in accommodative facility (Jaiswal et al., 2019).

The previous studies did not compare the effect on response times and also did not compare for different durations of near task simultaneously. Thus far, no study has compared accommodative facility and response times for two different durations in young adults simultaneously. It can also be noted that the minimum duration tested for most of the above studies is 30 minutes. Several studies have shown one hour of electronic devices usage increases visual symptoms in young adults by as much as five times (Jaiswal et al., 2019). However, 30 minutes is the smallest near task duration that had an effect on accommodative parameters, and one hour is the longest task duration that showed a significant influence on visual symptoms (Jaiswal et al., 2019). We were interested in seeing how two different durations of computer work sessions—30 minutes and one hour—would affect accommodative facility and response times. Hence, this study aimed to assess the impact of 30 minutes and one hour of computer work (laptop device) on accommodative facility and response times on the same set of participants, which has not been done previously.

## Methods

This was an experimental study in which 50 young adults (37 females and 13 males) participated. Sample size was calculated using G*Power (version 3.1.9.4) for a one side *p* value of 0.05, a power of 95%, and an effect size of 0.5. Myopic and non-myopic young adults aged 18–26 years with a visual acuity of 6/6 or better either with their habitual spectacles or unaided were included in the study. Participants with binocular vision anomalies and ocular pathologies were excluded from the study. However, participants who had poor accommodative facility <6 cycles per minute were included in the study to observe how the near task may affect those with accommodative infacility.

This study adhered to the Tenets of the Declaration of Helsinki. The study was approved by the Ethics Committee of SRM Medical College Hospital and Research Centre. Written, informed consent was obtained from all participants before starting measurements.

A complete ocular history that included details about their symptoms, past medical and ocular history was obtained from all participants. All participants were screened for visual acuity with current prescription. Refractive status was measured using Shin-Nippon NVision-K 5001 Openfield autorefractor without cycloplegia. Myopia was defined as spherical equivalent ≤–0.50 D (Pandian et al., 2006). Emmetropia was defined as spherical equivalent >–0.50 D, but <1.50 D (Pandian et al., 2006). A +1.00 D blur-test, which reduced acuity to less than 6/9, was performed to exclude those with uncorrected hyperopia. All participants underwent visual examination (Table 1), and those with findings outside prescribed normative range (Scheiman & Wick, 2014) were excluded from the study.

**Table 1:** Tests performed for all the participants.


TEST PARAMETER	PARAMETER MEASURED USING

Stereopsis	Randot stereotest

Heterophoria	Cover test Modified Thorington

Suppression	Modified Thorington (each participant was asked if both the streak and the spotlight were visible to them before starting the test)

AC/A	Calculated method using the formula: Distance IPD + 0.4 (near phoria – distance phoria)

Near point convergence	Accommodative target: linear target (6/6) Non-accommodative target: red-green goggles and penlight

Fusional vergence (NFV and PFV)	Horizontal prisms

Vergence facility	12ΔBO/3ΔBI vergence flippers

Near point of accommodation (monocular and binocular)	Push up test (continuous text chart and N6 target)

Accommodative facility (monocular and binocular)	Accommodative flippers of +2.00 DS/–2.00 DS


### Pilot study

The purpose of the pilot was to only elicit the time taken to flip the lenses by the examiner and not the time taken to respond to ±2.00 DS flipper. A trial run was conducted using a plano flipper to measure the operator delay for flipping the lenses. Plano flipper was flipped at the maximum possible speed for a period of 30 seconds for five participants. The time taken for the operator to flip the lenses was calculated as 0.4 seconds, indicating that for each cycle, only 0.8 seconds were contributed to operator delay and time taken to flip the lens. This was deemed a negligible amount because a dummy run done by Radhakrishnan et al. had similar results with an operator delay of 0.6 seconds (Radhakrishnan, Allen & Charman, 2007).

### Accommodative facility testing

**Figure 1:**
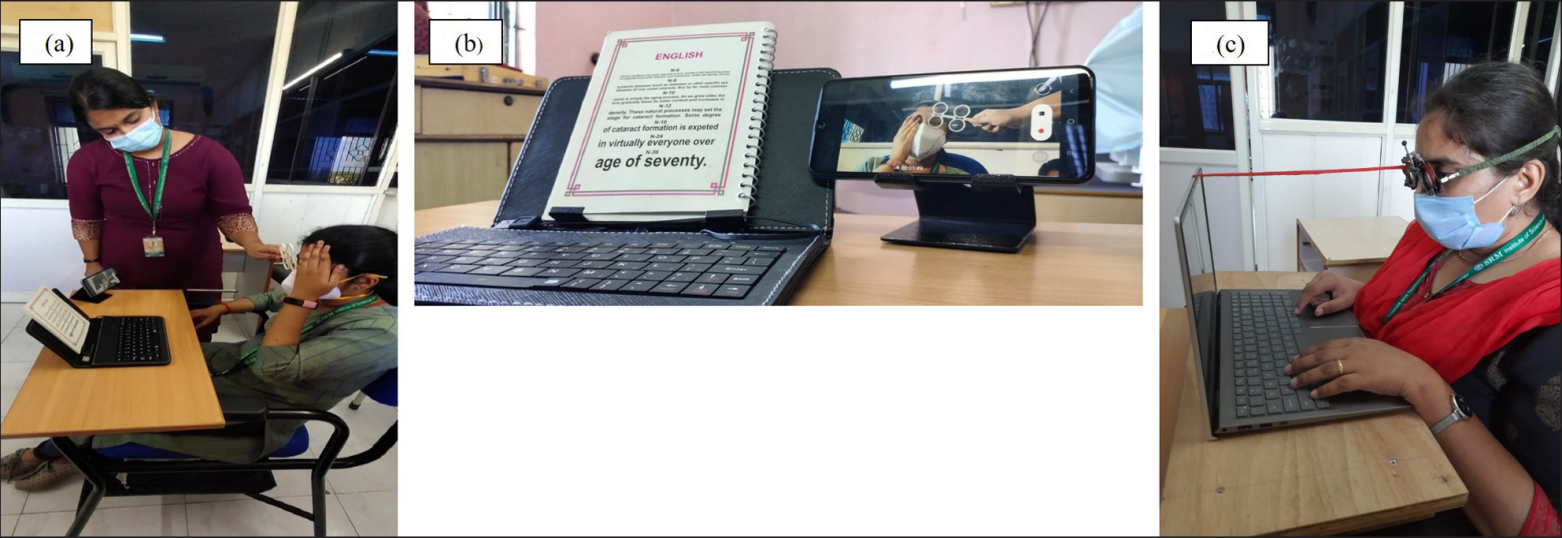
**(a)** Image showing pre and post task setup. **(b)** Image showing N6 target given for pre and post task and simultaneous video recording through smartphone. **(c)** Image showing reading task on the laptop, –2.00 D induced in front of both eyes of the participant and 25cm working distance maintained throughout the task.

Participants were asked to close their eyes for 15 minutes in a dark room before pre-task measurement to eliminate any prior near work aftereffect. Testing was done with habitual refractive correction. Monocular near accommodative facility was measured using ±2.00 DS flipper at 40 cm using the N6 target before and after two reading tasks (Figure 1a). To avoid convergence interaction, only monocular accommodative facility was tested. Because there was a high correlation between right eye and left eye measurements during visual examination, for monocular accommodative facility, each participant had either their right eye or left eye tested. The order of testing was randomised. The other eye was occluded during testing. Each flip constituted a half cycle, and two consecutive flips was a full cycle. A facility of zero was recorded when no response was made by the participant in one minute. Response time was recorded as 60 seconds for such participants. The second task was done on a different day with a time gap of one to three weeks between the two tasks.

Participants were instructed as follows:

Focus on the smallest row of letters and make sure it is clear. One side of the lens will be placed in front of your eye, and the print will initially be blurred for a brief period of time and then become clear. The moment the print becomes clear, you should say ‘clear’. The lens will be flipped to the other side, and the print will appear blurred again; say ‘clear’ once the print becomes clear again. The flipping of the lens back and forth will be repeated for 1 minute.

Both pre- and post-task testing were video recorded using a smart phone for later analysis. (Figure 1b). The number of cycles per minute and positive and negative response times were calculated through the video recording. Response time was recorded as the number of seconds taken to clear the lens kept in front of the eye. Positive response time was recorded as the time taken to clear the negative lens, and negative response time was recorded as the time taken to clear positive lens. The mean positive and negative response times were calculated for each participant. The pre- and post-task values were recorded in separate files and also analysed separately.

**Task 1:** Participants were asked to read *Harry Potter and the Sorcerer’s Stone* on a laptop for 30 minutes while viewing through a –2.00 D lens. The –2.00 D lens was for increasing the accommodative demand during the computer work. The distance between the laptop and participant’s eyes was 25 cm, which remained constant throughout the computer work (Figure 1c). Therefore, an accommodative demand of 6 D came into play throughout the computer work. The target size was N12 with a visual angle of 0.69° or 0.11 radians at 25 cm. The laptop screen display was a 15.6-inch diagonal LED-backlit HD anti-glare (1366 × 768 resolution) with full brightness level on the screen. The laptop screen dimensions were 14.64 × 9.83 × 1.09 inches.

**Task 2:** Participants were asked to read *Harry Potter and the Sorcerer’s Stone* on the same laptop used in Task 1 for one hour while viewing through a –2.00 D lens. The working distance and accommodative demand and working environment were all identical to that of Task 1. The order of task was randomised.

## Statistical Analysis

Statistical analysis was carried out with SPSS Version 22.0. Data on monocular near accommodative facility rate and positive and negative response times, both pre- and post-tasks, were tested for normality using Kolmogorov Smirnov normality test. Excluding pre-task and post-one-hour accommodative facility, all the other parameters were not normally distributed. Hence non-parametric tests were performed. The mean pre- and post-task measures were compared using the Wilcoxon Signed Ranked test to analyse the effect of 30-minute and 1-hour durations of computer work on accommodative facility rates and positive and negative response times in young adults.

A mixed between-within subjects analysis of variance was conducted to assess the impact of task duration on accommodative facility and response times between refractive groups (myopes and emmetropes) across three time points (baseline, after the 30-minute task, after the 1-hour task).

## Results

**Figure 2:**
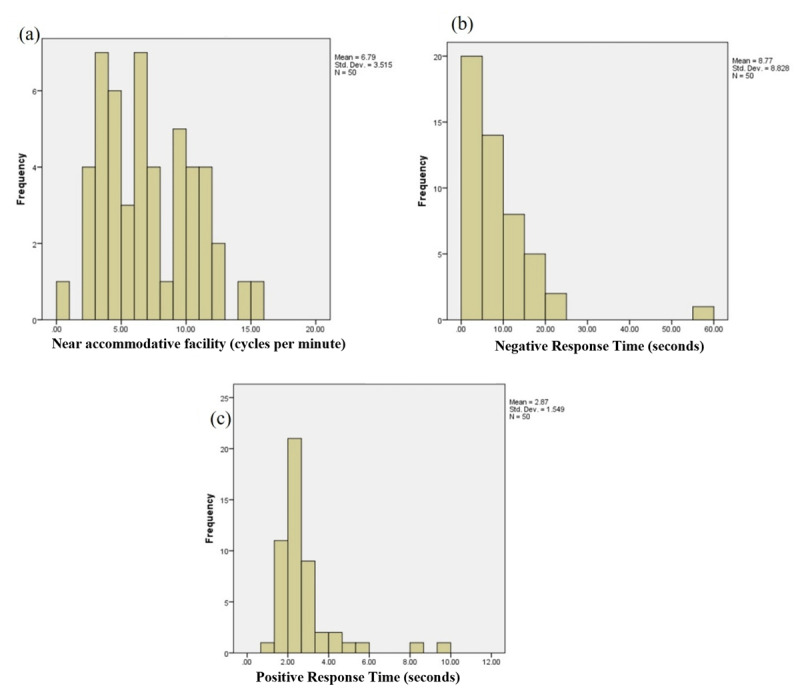
**(a)** Frequency distribution of the mean near accommodative facility for the 50 participants. **(b)** Frequency distribution of the mean negative response time for the 50 participants. **(c)** Frequency distribution of the mean positive response time for the 50 participants.

Out of 60 participants, 4 dropped out and 6 did not meet the eligibility criteria. Data of 50 participants (29 emmetropes and 21 myopes) were analysed. The mean age of the participants was 20.68 ± 1.33 years. The mean spherical equivalent refractive error of emmetropes was 0.16 ± 0.28 D for the right eye and 0.17 ± 0.33 D for the left eye. The mean spherical equivalent refractive error of myopes was –1.95 ± 1.14 D for the right eye and –1.83 ± 1.23 D for the left eye. The mean monocular near accommodative facility for this clinical sample was 10.13 ± 3.97 cycles per minute for the right eye and 10.38 ± 4.11 cycles per minute for the left eye. Figure 2a, b, and c depict the frequency distribution of monocular near accommodative facilities, negative response time, and positive response time, respectively, for this clinical sample.

Because there were 21 participants with accommodative facility poorer than the normative range of 6–16 cycles per minute (Scheiman & Wick, 2014), data were also analysed separately for participants with accommodative facility > 6 cycles per minute and for those with < 6 cycles per minute (Table 2).

**Table 2:** Comparison of baseline characteristics between normal accommodative facility group (≥ 6 cpm) and reduced accommodative facility group (<6 cpm).


CHARACTERISTICS		NORMAL ACCOMMODATIVE FACILITY GROUP (≥6 CPM), MEAN ± SD	REDUCED ACCOMMODATIVE FACILITY GROUP (<6 CPM), MEAN ± SD	*P* VALUE

Stereopsis (arc seconds)		33.62 ± 9.15	31.43 ± 9.10	0.41

Heterophoria (PD)	Distance	0.03 ± 0.19	0.02 ± 0.84	0.95

	Near	–1.09 ± 1.99	–0.95 ± 2.31	0.83

AC/A		5.46 ± 0.87	5.53 ± 0.97	0.79

NPC (accommodative target) (cm)	Break	7.00 ± 3.06	6.98 ± 3.87	0.98

	Recovery	9.62 ± 3.97	9.40 ± 4.17	0.85

NPC (non-accommodative target) (cm)	Break	8.86 ± 3.26	7.07 ± 3.26	0.61

	Recovery	11.88 ± 3.76	9.74 ± 3.56	0.05

NFV (Distance) (PD)	Blur	0.69 ± 2.58	1.71 ± 3.70	0.28

	Break	9.14 ± 5.99	8.43 ± 4.80	0.66

	Recovery	6.76 ± 5.37	5.57 ± 4.27	0.41

NFV (Near) (PD)	Blur	2.28 ± 5.23	3.24 ± 5.95	0.55

	Break	13.41 ± 6.79	12.86 ± 5.81	0.76

	Recovery	10.69 ± 5.79	10.19 ± 5.06	0.75

PFV (Distance) (PD)	Blur	1.93 ± 4.58	7.43 ± 7.10	0.004

	Break	14.86 ± 7.18	17.52 ± 7.18	0.20

	Recovery	12.03 ± 6.05	13.95 ± 6.45	0.29

PFV (Near) (PD)	Blur	3.03 ± 6.22	4.57 ± 8.32	0.46

	Break	17.45 ± 7.94	20.90 ± 10.08	0.18

	Recovery	14.17 ± 6.85	16.52 ± 8.69	0.29

Vergence facility (cpm)		12.66 ± 2.17	11.57 ± 3.50	0.18

Amplitude of accommodation (D)	Right Eye	12.42 ± 3.12	14.12 ± 5.81	0.23

	Left Eye	13.39 ± 4.17	14.99 ± 6.98	0.36

	Both Eyes	15.56 ± 6.06	16.43 ± 8.91	0.68

Accommodative facility (cpm)	Right Eye	11.41 ± 3.71	8.36 ± 3.68	0.01

	Left Eye	11.86 ± 3.68	8.33 ± 3.85	0.002

	Both Eyes	11.16 ± 3.15	7.36 ± 3.90	< 0.001


In the present study, 31 out of 50 participants (62%) showed a reduction in near accommodative facility after 1 hour of computer work. Also, 32 out of 50 participants (64%) showed a delay in negative response time after 1 hour of computer work.

### Overall sample

**Figure 3:**
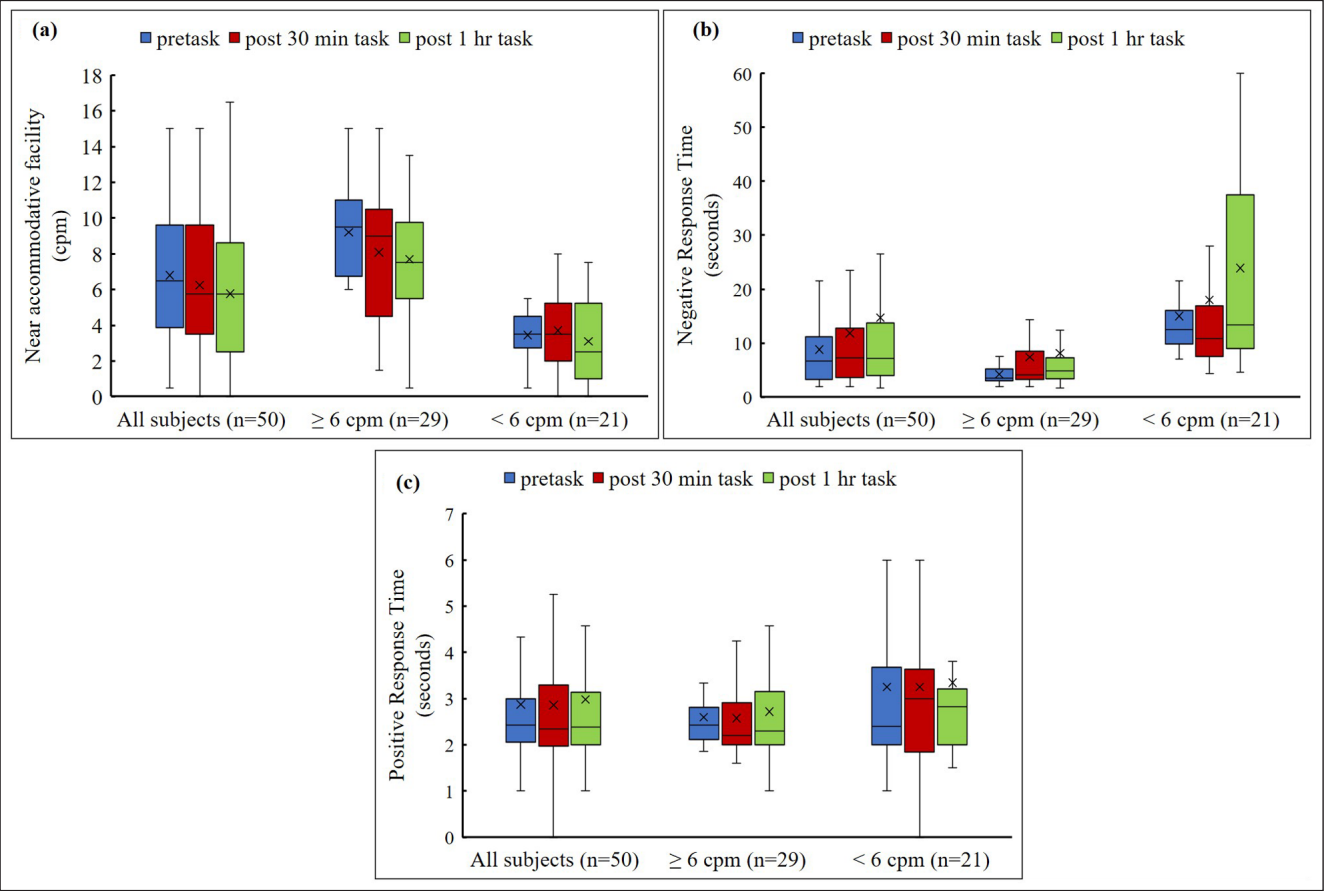
**(a)** Box & whisker plot comparing near accommodative facility pre and post 30 minutes and 1 hour of computer work. **(b)** Box & whisker plot comparing negative response time pre and post 30 minutes and 1 hour of computer work. **(c)** Box & whisker plot comparing positive response time pre and post 30 minutes and 1 hour of computer work. x – mean. ‘―’ – median.

In the overall sample of 50 participants, upon comparing before and after 30 minutes of computer work, results showed no significant difference in mean monocular near accommodative facility (pre-task: 6.79 ± 3.52 cycles per minute, after 30-minute task: 6.25 ± 3.65 cycles per minute; *p* = 0.10) (Figure 3a), mean negative response time (pre-task: 8.77 ± 8.83 seconds, after 30-minute task: 11.83 ± 14.28 seconds; *p* = 0.16) (Figure 3b), and positive response time (pre-task: 2.87 ± 1.55 seconds, after 30-minute task: 2.86 ± 1.67 seconds; *p* = 0.88) (Figure 3c). However, a significant decrease in mean monocular near accommodative facility (after 1-hour task: 5.76 ± 3.89 cycles per minute; *p* = 0.01) (Figure 3a) and a significant delay in mean negative response time (after 1-hour task: 14.72 ± 17.32 seconds; *p* = 0.03) (Figure 3b) was noted following the 1-hour computer task. There was no significant change observed in mean positive response time before and after 1 hour of computer work (after 1 hour task: 2.98 ± 2.33 seconds; *p* = 0.42) (Figure 3c). Effect size is an important indicator of clinical significance. For the present results, the effect size was 0.28, which is a small effect, indicating minimal clinical significance.

### Sample with normal accommodative facility

Among 29 participants with accommodative facility ≥ 6 cycles per minute, there was a statistically significant reduction in accommodative facility following both 30 minutes and 1 hour of computer work (pre-task: 9.21 ± 2.48 cycles per minute, after 30-minute task: 8.09 ± 3.40 cycles per minute, after 1-hour task: 7.69 ± 3.68 cycles per minute; *p* = 0.01), and a significant delay was observed in negative response time (pre-task: 4.23 ± 1.64 seconds, after 30-minute task: 7.36 ± 8.82 seconds, after 1-hour task: 8.10 ± 11.62 seconds; *p* = 0.004). There was no significant change observed in positive response time after 30 minutes and 1 hour of computer work (pre-task: 2.60 ± 0.69 seconds, after 30-minute task: 2.58 ± 0.97 seconds, after 1-hour task: 2.72 ± 1.16 seconds; *p* = 0.77).

### Sample with reduced accommodative facility

However, in the 21 participants with accommodative facility <6 cycles per minute, there was no statistically significant difference in accommodative facility after 30 minutes and 1 hour of computer work (pre-task: 3.45 ± 1.22 cycles per minute, after 30-minute task: 3.71 ± 2.19 cycles per minute; *p* = 0.49, after 1-hour task: 3.10 ± 2.28 cycles per minute; *p* = 0.57), negative response time (pre-task: 15.04 ± 10.78 seconds, after 30-minute task: 18.01 ± 17.94 seconds; *p* = 0.55, after 1-hour task: 23.88 ± 19.85 seconds; *p* = 0.38), and positive response time (pre-task: 3.25 ± 2.23 seconds, after 30-minute task: 3.25 ± 2.30 seconds; *p* = 0.79, after 1-hour task: 3.34 ± 3.35 seconds; *p* = 0.56).

The pre- and post-task accommodative facility measurements did not differ significantly between the refractive groups—myopes and emmetropes (F_(2, 47)_ = 0.95 *p* = 0.39). The pre- and post-task negative response time measurements did not differ significantly between the refractive groups—myopes and emmetropes (F_(2, 47)_ = 0.56; *p* = 0.57). The pre- and post-task positive response time measurements did not differ significantly between the refractive groups—myopes and emmetropes (F_(2, 47)_ = 3.23; *p* = 0.05).

## Discussion

The results show that there was a significant reduction in monocular near accommodative facility and a significant delay in negative response time following 1 hour of computer work in the overall sample. Operator delay in flipping the lens is less likely to account for these differences because the trial run revealed 0.8 seconds to flip the lens, which is negligible. The reduction in accommodative facility cycles is due to a delay in relaxing accommodation through positive lenses. The results of the present study are in agreement with the findings of Jiang and White (1999), who also found a decrease in monocular near accommodative facility after computer work and an increase in the duration for relaxing accommodation, but not for stimulating accommodation. However, unlike Jiang and White (1999), who found a decrease in accommodative facility and delay in negative response time after 20 minutes of computer work, the difference was observed only after 1 hour of computer work. Although both studies kept an accommodative demand of 6 D during the near task, in the current study, the near task was to read a story on a personal computer whereas, in the previous study participants played an interactive computer game on a computer monitor (positioned at 50 cm and viewed through a –4 D lens). The lenses were flipped by the examiner in the present study, whereas the participants flipped the lenses in the previous study. The response times were calculated by the computer because they had used a semi-automated flipper, whereas the present study had used a manual flipper and calculated response times manually through the video recording. In the previous study, the experiment was repeated three times over a period of one to two weeks, and data were averaged across the three sessions, whereas in the present study, each of the test procedures was done in one attempt alone.

Both near accommodative facility and negative response time were not significantly affected after 30 minutes of computer work. Positive response time was not delayed following either 1 hour or 30 minutes of computer work.

In the overall sample, 21 participants’ accommodative facility rate was < 6 cycles per minute, and 29 had an accommodative facility rate ≥ 6 cycles per minute. When the 29 participants were taken into consideration, there was a significant decrease in accommodative facility and a significant delay in negative response time after 30 minutes and after 1 hour of computer work. Accommodative facility rates declined after computer work in participants having normal accommodative facility prior to computer work, indicating that accommodative facility becomes poorer after extensive computer work and might lead to accommodative infacility. However, when looking at the data of the 21 participants with poorer facility prior to the task, there was no significant difference seen in accommodative facility and negative response time after 30 minutes and 1 hour of computer work. Although negative response time showed a 9-second delay following 1 hour of computer task in 21 participants, it did not reach statistical significance, but 4 seconds of delay in 29 participants was statistically significant. This could be due to the abnormal distribution of data points. Outliers can have a significant impact on statistical analysis and skew the results. Outliers may also influence statistical power, which makes it difficult to detect a true effect if there is one. There is a possibility that the pre-task facility testing may have had a training effect for the 21 participants, thus resulting in statistical insignificance. There was no significant change observed in positive response time after both tasks in either group, indicating that positive response time is seldom affected by near task. This is similar to an earlier study, which did not report changes in positive response time following 20 minutes of computer work (Jiang & White, 1999).

The present study reported a significant decrease in monocular accommodative facility after the 1-hour task by 6% of the baseline value, which is about one cycle. The negative response time was also significantly delayed by about 77% of the baseline value, which is about 6 seconds, after 1 hour of computer work. This magnitude of change, in particular for the negative response time, is quite alarming because it indicates that participants had difficulty in relaxing accommodation, possibly due to near work–induced transient effect. The positive response times were faster by 4–6% and from baseline after the 1-hour and 30-minute durations, accounting for 0.1 seconds, but this was not significant. After the 30-minute task, monocular accommodative facility dropped by 2% from baseline and the negative response time was prolonged by 48% from baseline, but none of these changes were significant.

The finding that near accommodative facility did not show significant difference after 30 minutes of computer work is in agreement with Park et al. (2014), who also showed no significant change after 30 minutes of smartphone viewing. In addition, the result that accommodative facility reduced after 1 hour of computer work is in concordance with Golebiowski et al. (2020), who found a decrease in accommodative facility after 1 hour of reading on a smartphone. So regardless of the electronic device used, the present study’s results are in agreement with previous studies: after 1 hour of electronic device usage, there is significant reduction in accommodative facility, but this change is not seen after 30 minutes of using an electronic device. Despite different reading durations, the present study’s results also showed a decrease in accommodative facility.

In the present study, monocular accommodative facility reduced by about half a cycle after 30 minutes of computer work, which is similar to Park et al. (2014), who had a similar result. But the monocular accommodative facility reduced by 1 cycle after 1 hour of computer reading, which is much lower than that reported by Golebiowski et al. (2020), who showed a 3.5-cycle reduction after 1 hour of smartphone viewing, and Padavettan et al. (2021) showed a decrease of 2 cycles after 30 minutes of smartphone reading in binocular accommodative facility. Jiang and White (1999) showed a 2.5-cycle reduction in emmetropes, whereas myopes had only a 1-cycle reduction after 20 minutes of computer work. Although the present study results cannot be directly compared to the results reported by Jiang and White (1999), it can be seen that the higher the task durations, the higher the delay in negative response time will be.

With regard to refractive group difference, sustained computer work had a similar impact on accommodative facility and response times in both myopes and emmetropes across two time points. These results suggest that accommodative facility and response times after sustained computer work did not show differential refractive group susceptibility. This is in concordance with results found by Jiang and White (1999), who also did not report refractive group differences.

There are a few limitations in this study. The principal investigator was not masked even though pre- and post-task measurements were stored in separate files and pre-task values were analysed first followed by post-tasks values. This could have created a bias in the results. In addition, administering a symptom questionnaire immediately after each computer task could have been useful to identify which task duration had a greater impact on visual symptoms. Another limitation was that refractive error was not measured with cycloplegia for the 50 participants included in the study. If accommodative rock was used, the results obtained would be different from the present study, because lenses are not used in accommodative rock. The testing distance would also be different because the distance Hart chart would be kept at 3 metres, and the near Hart chart would be held at 40 centimetres. The results of the present study would not be the same if distance accommodative facility measurements were taken because the testing distance would change to 3 metres, and the flipper used would also differ; a plano/–2.00 D flipper would have been used. The results of this study pertain to the usage of a laptop, and this observation may be different depending on the size and letter size of the e-device and the distance between screen and eyes.

With increasing usage of personal computers and the gradual decline in the use of print material, a printed paper task was not included in this clinical sample. This is another limitation for the present study. However, previous studies conducted with printed paper versus text viewed on electronic devices found a more profound effect on accommodative parameters in text viewed on electronic devices than printed paper. We cannot generalise the results of the present study with paper task because we did not have a control group. However, in two previous studies, monocular accommodative facility was not significantly different after 30 minutes of reading from printed text. The transient effect could be a part of DES because previous studies show a significant difference after e-devices usage but not after paper near work.

To conclude, in the clinical sample examined in this study, monocular near accommodative facility significantly reduced in the young adults after one hour of computer work. Negative response time (time taken to relax accommodation) was delayed after one hour of computer work. Monocular near accommodative facility and negative response time had no significant change after 30 minutes of computer work. Positive response time (time taken to stimulate accommodation) also did not have any significant change after 30 minutes and 1 hour of computer work. Prolonged exposure to electronic devices is the same as continuous use when digital strain sets in. Sustained exposure to computers may later cause accommodative infacility in young adults having accommodative facility within the normative range.

## Clinical Implications

The results from this study will help develop guidelines for the use of e-devices. We would like to advocate that after every 30 minutes of computer work, one should take a break from the screen to maintain good eye health; one should also try to limit the use of overall screen exposure. The results from this study will help eye care professionals understand the impact of computer work of two different reading durations on accommodative facility rates and response times in young adults so as to plan and implement appropriate vision therapy for susceptible young adults.

## Future Research

Further studies including other accommodative or vergence parameters change after near task of durations longer than one hour in different refractive groups (hyperopes, myopes, and emmetropes) with different electronic devices and different language texts could be explored, because text-based differences in accommodative responses have been reported between Chinese text and English text (Yeo, Atchison & Schmid, 2013).
